# ALK5 signaling pathway mediates neurogenesis and functional recovery after cerebral ischemia/reperfusion in rats via Gadd45b

**DOI:** 10.1038/s41419-019-1596-z

**Published:** 2019-05-01

**Authors:** Keming Zhang, Qinbin Zhang, Jing Deng, Jinfang Li, Jiani Li, Lan Wen, Jingxi Ma, Changqing Li

**Affiliations:** 1 Department of Neurology, The Second Affiliated Hospital of Chongqing Medical University, Chongqing, China; 2 Department of Neurology, Chongqing General Hospital, Chongqing, China; 3 Department of Neurology, The Southwest Hospital, Chongqing, China

**Keywords:** Adult neurogenesis, Regeneration and repair in the nervous system, Brain injuries, Stroke, Stroke

## Abstract

Transforming growth factor β (TGF-β) serves critical functions in brain injury, especially in cerebral ischemia; however, apart from its neuroprotective effects, its role in regulating neurogenesis is unclear. TGF-β acts in different ways; the most important, canonical TGF-β activity involves TGF-β receptor I (TβRI) or the activin receptor-like kinase 5 (ALK5) signaling pathway. ALK5 signaling is a major determinant of adult neurogenesis. In our previous studies, growth arrest and DNA damage protein 45b (Gadd45b) mediated axonal plasticity after stroke. Here, we hypothesized that ALK5 signaling regulates neural plasticity and neurological function recovery after cerebral ischemia/reperfusion (I/R) via Gadd45b. First, ALK5 expression was significantly increased in middle cerebral artery occlusion/reperfusion (MCAO/R) rats. Then, we knocked down or overexpressed ALK5 with lentivirus (LV) in vivo. ALK5 knockdown reduced axonal and dendritic plasticity, with a concomitant decrease in neurological function recovery. Conversely, ALK5 overexpression significantly increased neurogenesis as well as functional recovery. Furthermore, ALK5 mediated Gadd45b protein levels by regulating Smad2/3 phosphorylation. Finally, ALK5 coimmunoprecipitated with Gadd45b. Our results suggested that the ALK5 signaling pathway plays a critical role in mediating neural plasticity and neurological function recovery via Gadd45b after cerebral ischemia, representing a new potential target for cerebral I/R injury.

## Introduction

Stroke is one of the leading causes of disability in human adults and accounts for 44 million disabilities worldwide each year^1^. Approximately 80–90% of all stroke cases are ischemic stroke, while 10–15% are hemorrhagic stroke^2^. At present, the most effective treatment for ischemic stroke is rapid restoration of the blood supply. However, blood reperfusion may aggravate brain injury and functional impairment^3^. The process of restoring blood flow and causing reperfusion injury after cerebral ischemia is called cerebral ischemia/reperfusion (I/R) injury. The mechanisms of cerebral I/R injury are complex, with multiple signaling pathways and biological processes involved^4,5^. Improving functional recovery after cerebral I/R injury is an urgent key problem that must be solved. However, information about the mechanisms of injury and protection to brain tissues after cerebral I/R remains limited, and there are no satisfactory therapeutic strategies. According to an increasing number of studies, enhancing brain plasticity and promoting neurogenesis after stroke can significantly improve functional recovery^2,6–8^.

Transforming growth factor-β (TGF-β) is a multifunctional secreted cytokine that plays a crucial role in cell proliferation, differentiation and apoptosis^9^. According to many studies, TGF-β has extensive neuroprotective functions^10,11^. Activin receptor-like kinase 5 (ALK5), also known as TGF-β type I receptor (TβRI), plays a key role in the canonical TGF-β signaling pathway. ALK5 can catalyze the phosphorylation of receptor-regulated Smad proteins (i.e., Smad2 and Smad3), which form a heteromeric complex with common-mediated Smad4 and translocate into the nucleus where they regulate transcription^9,12^. Based on recent studies, the ALK5-dependent TGF-β signaling pathway is involved in neurogenesis^12,13^. However, the mechanisms of the ALK5 signaling pathway that promote neurogenesis and whether ALK5 is involved in neurogenesis after cerebral ischemia remain unclear.

Growth arrest and DNA damage protein 45b (Gadd45b), a member of the GADD45 family, plays a key role in anti-apoptosis and DNA repair^14,15^. We previously demonstrated that Gadd45b can improve axonal plasticity and functional recovery after stroke^7^. In addition, our studies indicated that Gadd45b is a critical mediator of neuronal apoptosis and autophagy^5,16^. Another study demonstrated that Gadd45b can promote adult neurogenesis^17^. According to an increasing number of studies, ALK5 signaling can regulate the expression of Gadd45b^15,18,19^. Based on all these findings, we hypothesize that after cerebral I/R injury, ALK5 signaling can promote neurogenesis through GADD45b, thereby promoting functional recovery.

To verify our hypothesis, we first employed a MCAO/R rat model and used anterograde neuronal tracer biotinylated dextran amine (BDA), Golgi staining and immunohistochemical staining of neurofilament protein 200 (NF-200) to demonstrate that ALK5 promotes neurogenesis. Moreover, ALK5 improved functional recovery after cerebral I/R injury. Furthermore, the ALK5/Smad2/Smad3 signaling pathway served as an upstream regulator of Gadd45b expression, and ALK5 increased Gadd45b expression by promoting Smad2/3 phosphorylation. Finally, ALK5 coimmunoprecipitated with Gadd45b.

## Materials and methods

### Experimental animals

Healthy adult male Sprague-Dawley rats (200–250 g) were purchased from the Experimental Animal Centre of Chongqing Medical University (Chongqing, China). All rats were housed at a 12 h/12 h light/dark cycle room maintained at 21–22 °C with a relative humidity of 60% and allowed free access to food and water. All animal experimental procedures were performed in accordance with the National Institutes of Health Guide for the Care and Use of Laboratory Animals and were approved by the Ethics Committee of Chongqing Medical University.

### Right middle cerebral artery occlusion/reperfusion (MCAO/R) model

Right MCAO/R was performed as previously described^7^. Briefly, the common carotid artery, external carotid artery (ECA) and internal carotid artery (ICA) were exposed. A nylon filament suture rounded by paraffin wax at the tip was advanced from the ECA into the ICA. After 2 h of occlusion, the filament was withdrawn for blood reperfusion. Rats that failed to exhibit neurological deficits after reperfusion (neurological score < 1 or >3) as evaluated by the Longa-Z method^20^ were excluded from the study.

### Lentivirus administration

To knock down the expression of ALK5, we used a small interfering RNA (siRNA) with the sequence GGACCAAGCCTATGATGATAA. Lentiviral vector: psi-LVUGP, Promoter: U6, Reporter: EGFP, Resistance marker: Puromycin resistance gene. Lentiviral vectors expressing ALK5-RNAi (LV-ALK5-RNAi, 3.02 × 10^8^ TU/ml) were supplied by GeneCopoeia Inc. (Rockville, Maryland, USA). Lentiviral vectors coding for GFP were used as the control (LV-con-RNAi).

Lentiviral vectors for overexpressing ALK5 (LV-ALK5, 2.46 × 10^8^ TU/ml) were also supplied by GeneCopoeia Inc. Lentiviral vector: pEZ-Lv201, Promoter: CMV, Reporter: EGFP, Resistance marker: Puromycin resistance gene. Lentiviral vectors coding for GFP were used as the control (LV-con-ALK5).

Fourteen days before MCAO/R, lentiviruses were stereotaxically injected into the ischemic cortex at two sites on the right cortex as follows: A-P 1.0 mm, M-L −2.0 mm, D-V −1.2 mm; A-P −3.0 mm, M-L −1.5 mm, D-V −1.2 mm; 2.5 μl for each site^21^. The injection rate was 0.2 μl/min, and at the end of the injection, the microinjector was left in place for 5 min before withdrawal.

### Neurobehavioral assessment

The Modified Neurological Severity Score (mNSS) and adhesive-removal somatosensory test were performed 3 d before and 1, 7 and 14 d after MCAO/R.

The mNSS is a motor, sensory, reflex, and balance composite test. Neurological function was graded from 0 to 18 (normal score, 0; maximal deficit score, 18). In the severity scores of injury, 1 point indicates the inability to perform the test or the lack of a tested reflex^22^.

An adhesive-removal somatosensory test was used to evaluate sensorimotor deficits. Two pieces of 113.1 mm^2^ adhesive-backed papers were used as bilateral tactile stimuli that were adhered to the distal-radial region of each forelimb. The time to remove each stimulus was recorded during 3 trials per day (maximum limit: 120 s). Individual trials were separated by at least 5 min. Before MCAO/R, the animals should be trained for 3 d^23^.

### Western blot assay

Protein was extracted from ischemic hemisphere brain tissues from different groups using a whole protein extraction kit (Beyotime, China). The protein concentration was measured by BCA protein assay reagent (P0010S, Beyotime, China). After denaturation by boiling, all protein samples were stored at −80 °C until analysis. Equal amounts of protein were loaded into each well of SDS-PAGE gels, electrophoresed on 10% or 12% separating gels and transferred onto a PVDF membrane (IPVH00010, Millipore, USA). The membranes were blocked with 5% skim milk and incubated overnight at 4 °C with the following primary antibodies: anti-ALK5 (1:400, ab31013, Abcam, USA), anti-Smad2/3 (1:1000, 8685, Cell Signaling Technology, USA), anti-phospho-Smad2/3 (1:1000, 8828, Cell Signaling Technology, USA), anti-Gadd45b (1:1000, ARP48346_P050, Aviva Systems Biology, USA), anti-growth-associated protein 43 (GAP-43) (1:200, sc-33705, Santa Cruz Biotechnology, USA), and anti-GAPDH (1:4000, 10494-1-AP, Proteintech, China) antibodies. GAPDH was used as a loading control. After washing with TBST, the membranes were incubated with horseradish peroxidase-conjugated goat anti-rabbit IgG antibody (1:4000, SA00001-2, Proteintech, China) or anti-mouse IgG antibody (1:4000, SA00001-1, Proteintech, China) for 1 h at room temperature. Then, the membranes were washed with TBST again and visualized using an enhanced chemiluminescence (ECL) reagent (P0018FS, Beyotime, China). Images were captured by a Fusion FX5 analysis system (Vilber Lourmat, F-77601 Marne-la-Vallée cedex 3, France) and quantified using Quantity One software (Bio-Rad Laboratories, USA).

### Immunohistochemistry

Animals were anesthetized at 24 h and 14 d after MCAO/R and fixed through the left ventricle with PBS followed by 4% paraformaldehyde. Five slides were randomly selected from each sample for analysis. Sections were deparaffinized in xylene, rehydrated in graded ethanol solutions, placed in 10 mM sodium citrate buffer (pH 6.0) and heated in a microwave for antigen retrieval. Endogenous peroxidase activity was blocked with 3% H_2_O_2_ for 15 min at 37 °C. Next, the sections were blocked with normal goat serum for 30 min at 37 °C. Then, the sections were incubated overnight at 4 °C with the following primary antibodies: anti-ALK5 (1:50, ab31013, Abcam, USA) and anti-neurofilament-200 (1:50, sc-32729, Santa Cruz Biotechnology, USA) antibodies. Next, the sections were incubated with goat anti-rabbit IgG or goat anti-mouse IgG (Zhongshan Golden Bridge Inc., China) for 30 min at 37 °C. Positive activity was revealed by 3-3′diaminobenzidine (DAB) (Zhongshan Golden Bridge Inc., China). Images were captured by a LEICA DM600B automatic microscope (Leica Microsystems Heidelberg GmbH, Germany) and quantified using Image-Pro Plus 6.0 software (Media Cybernetics, USA). Protein expression levels were reflected by the mean optical density value (integrated optical density (IOD) divided by the relevant area).

### Immunofluorescence

Brain tissues were cut into frozen sections. The frozen sections were air-dried at room temperature, washed with PBS, permeabilized with 0.4% Triton X-100, and submitted to antigen retrieval as for immunohistochemistry. After washing with PBS, sections were blocked in normal goat serum for 1 h. Then, the sections were incubated overnight at 4 °C with the following primary antibodies: anti-ALK5 (1:50, SAB4502958, Sigma-Aldrich, USA), anti-neuron-specific beta-III tubulin (1:50, MAB1195, R&D, USA), anti-GFAP (1:100, BM0055, Boster, China), anti-nestin (1:100, ab11306, Abcam, USA), and anti-DCX (1:50, sc-271390, Santa Cruz Biotechnology, USA) antibodies. The next day, the sections were washed with PBS and incubated with a mixture of goat anti-rabbit IgG-CFL 488 (1:100, sc-362262, Santa Cruz Biotechnology, USA) and goat anti-mouse IgG-CFL 555 (1:200, sc-362267, Santa Cruz Biotechnology, USA) at 37 °C for 1 h in the dark. Then, the sections were counterstained with DAPI for nuclei. Images were captured by a confocal laser scanning microscope (A1 + R, Nikon, Tokyo, Japan).

### Biotinylated dextran amine (BDA) tracing

Four weeks after MCAO/R, 15 μl of a 10% BDA solution (BDA-10,000 Neuronal Tracer Kit, N7167, Invitrogen, USA) was injected into the left sensorimotor cortex at the following five coordinates: 1.0 mm rostral to bregma, 1.0 mm lateral to the midline, and 1.5 mm ventral to the dura; 4.0 mm caudal to bregma, 1.0 mm lateral to the midline, and 1.5 mm ventral to the dura; 1.0 mm rostral to bregma, 5.0 mm lateral to the midline, and 1.5 mm ventral to the dura; 4.0 mm caudal to bregma, 5.0 mm lateral to the midline, and 1.5 mm ventral to the dura; and 1.5 mm caudal to bregma, 2.5 mm lateral to the midline, and 1.5 mm ventral to the dura^7,21^.Two weeks after BDA injection, animals were transcardially perfused with PBS followed by 4% paraformaldehyde. The entire brains were removed, post-fixed overnight in 4% paraformaldehyde and dehydrated in graded sucrose solutions. Brain tissues were cut into 50 μm-thick coronal sections by a cryostat microtome (Leica CM1950, Germany). All sections were collected in PBS, incubated with 0.5% H_2_O_2_ for 20 min, and submitted to 0.5% Triton X-100 for 30 min. Then, the sections were incubated with avidin–biotin–peroxidase complex (Invitrogen, USA) overnight at room temperature and DAB (Invitrogen, USA) for 5 min^7,8^.

The sections were examined using a ×10 objective. Then, we counted individual axons under a ×20 objective. The fibers projecting to the right red nucleus were examined by counting all midline-crossing BDA-positive fibers. The number of midline-crossing BDA-positive fibers was divided by the total number of corticospinal tract fibers^7,21^. The method of counting positive fibers crossing the midline of the corpus callosum was similar to that of the red nucleus. BDA-positive fibers were counted using Image-Pro Plus 6.0 software.

### Golgi–Cox staining

Golgi–Cox staining was conducted according to the manufacturer’s instructions for a HITO GOLGI-COX OPTIMSTAIN™ KIT (Hitobiotec Corp., USA). Briefly, whole brain tissues were removed rapidly and immersed into a mixture of solution 1 and solution 2 for 14 d in the dark and transferred to solution 3 for 24–72 h at 4 °C in the dark. Sections (200 μm-thick) were cut by a vibratome (Leica CM3050S, Germany). For staining, sections were mounted on 3% gelatin-coated glass slides, air-dried, stained with solutions 4 and 5, followed by graded ethanol solutions, dehydration and clearing with xylene. Layer III or V pyramidal neurons were identified with a ×20 objective and captured under a ×40 objective. A total of 20–25 neurons were measured for each group. The length and distribution of dendrites were evaluated by Sholl analysis^24^ as follows: 2D reconstructions of the entire dendritic tree were generated, a series of concentric circles with 10 μm intervals were centered on the soma, and the number of intersections was used to estimate the total length and distribution of dendrites^25–27^.

### Coimmunoprecipitation

Coimmunoprecipitation was conducted according to the manufacturer’s instructions for Protein A/G Magnetic Beads (HY-K0202, MedChemExpress, USA). Briefly, after washing the magnetic beads with binding/wash buffer, the beads were incubated with anti-ALK5 antibody (ab31013, Abcam, USA), anti-Gadd45b (ARP48346_P050, Aviva Systems Biology, USA) or anti-IgG antibody (ab172730, Abcam, USA) on the rotator for 2 h at 4 °C. Then, the supernatant was removed and discarded, the beads were washed, and protein samples were added and incubated with rotation for 2 h at 4 °C. After washing, the supernatant was discarded, and elution buffer was added. The supernatants were collected and used as samples for Western blotting. The antibodies used for Western blotting were as follows: anti-ALK5 (1:400, ab31013, Abcam, USA), anti-Gadd45b (1:800, ARP48346_P050, Aviva Systems Biology, USA), HRP-Mouse Anti-Rabbit IgG Light Chain Specific (1:4000, SA00001-7L, Proteintech, China) and horseradish peroxidase-conjugated goat anti-rabbit IgG antibody (1:4000, SA00001-2, Proteintech, China) antibodies.

### Statistical analysis

The data are presented as the means ± standard deviation (SD). SPSS 23.0 was used for statistical analyses, and GraphPad Prism 7.0 was used for graphing. Student’s t test, one-way or two-way analysis of variance (ANOVA) with a Bonferroni post hoc test were used to compare the differences between groups. *P* < 0.05 was considered statistically significant.

## Results

### Expression of ALK5 in a MCAO/R rat model

**Fig. 1:**
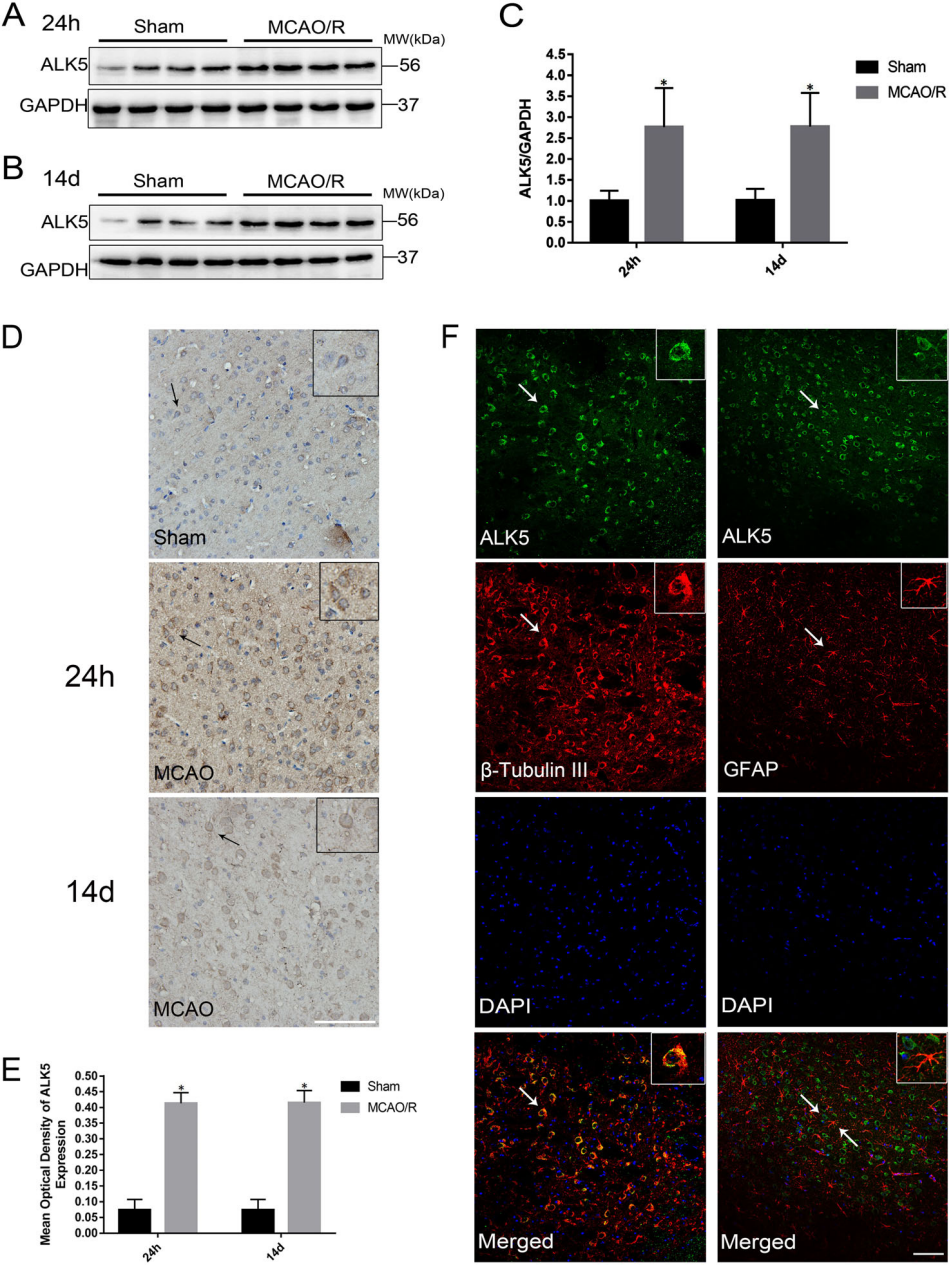
ALK5 is increased in the ischemic hemisphere in a MCAO/R rat model. **a** Representative images of ALK5 expression in the ischemic hemisphere 24 h after I/R (*n* = 6 biological replicates). **b** Representative images of ALK5 expression in the ischemic hemisphere at 14 d after I/R (*n* = 6 biological replicates). **c** Comparison of mean intensity ratios in Western blot analysis. **d** Representative images of immunohistochemical staining for ALK5 expression 24 h and 14 d after I/R (*n* = 5 biological replicates) (scale bar = 100 μm). **e** Comparison of the mean density value in immunohistochemical analysis for ALK5 expression. **f** Representative images of immunofluorescence staining for ALK5 (green), beta-III tubulin (red)/GFAP (red) and cellular nuclei (blue). (scale bar = 100 μm). Arrows show the positive cells, and the inserted images show magnified images of representative cells. *P < 0.05, compared to the sham group at the same time point (Student’s *t* test)

The expression and location of ALK5 were examined by Western blotting, immunohistochemistry and immunofluorescence. Western blotting showed that the expression of ALK5 was higher in the ischemic cortex of the MCAO/R group than in the sham group 24 h and 14 d after MCAO/R (*P* < 0.05, Fig. 1a–c). The results of immunohistochemistry were similar to those of Western blotting (*P* < 0.05, Fig. 1d, e). ALK5 was observed in the cytomembrane and cytoplasm and colocalized with the neuron marker β-III tubulin but not with the astrocyte marker GFAP (Fig. 1f).

### Expression of ALK5 after injection of recombinant lentivirus

**Fig. 2:**
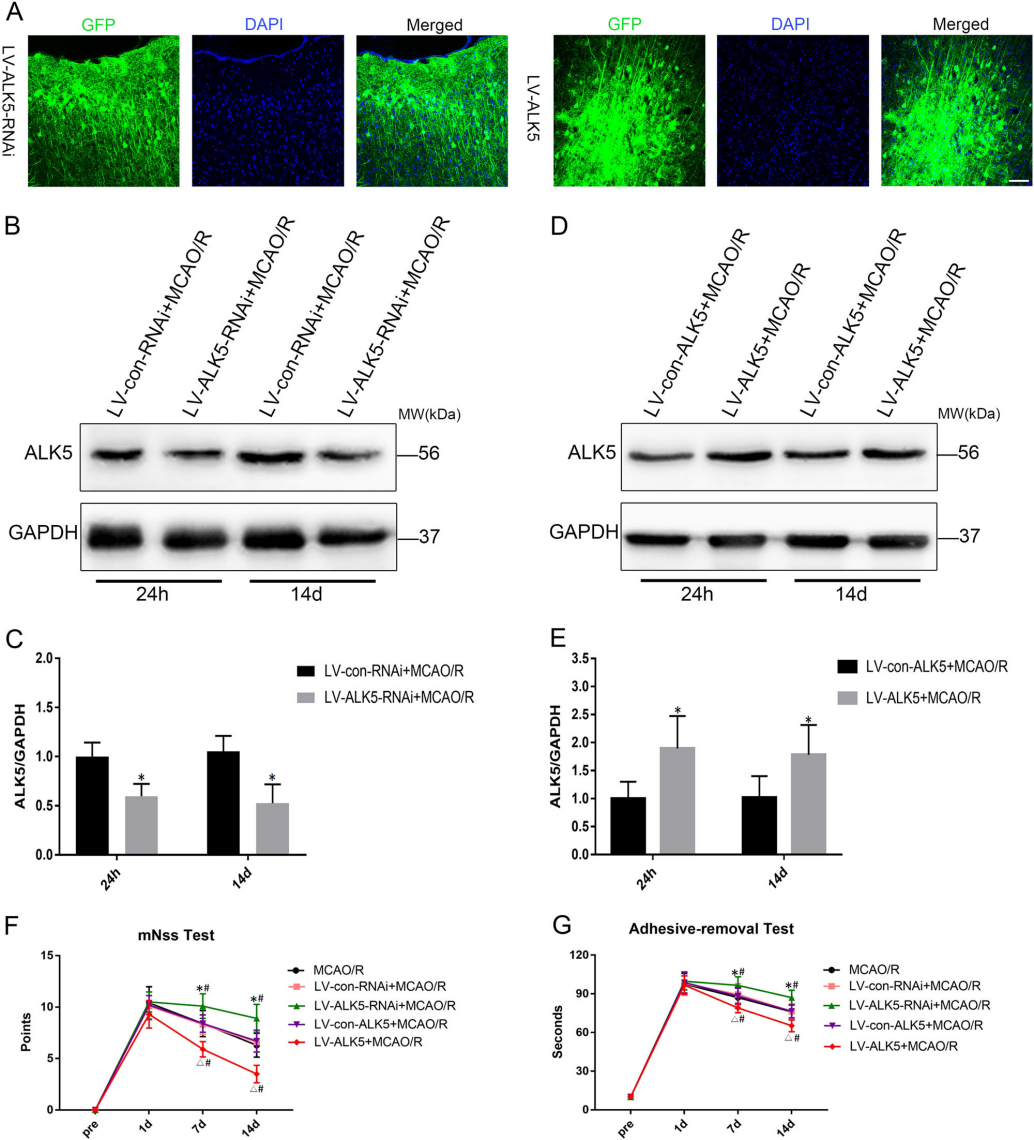
Lentiviruses are successfully transfected into neurons and effectively alter the expression of ALK5; ALK5 improves neurological function recovery. **a** Fluorescent images of GFP (green) in injected cortex 14 d after lentiviruses were stereotaxically injected (scale bar = 100 μm). **b** Representative Western blot images of ALK5 expression with LV-ALK5-RNAi or LV-con-RNAi injection 24 h and 14 d after I/R (*n* = 6 biological replicates). **c** Comparison of mean intensity ratios in Western blot analysis for ALK5 knockdown. **d** Representative Western blot images of ALK5 expression with LV-ALK5 or LV-con-ALK5 injection 24 h and 14 d after I/R (*n* = 6 biological replicates). **e** Comparison of mean intensity ratios in Western blot analysis for ALK5 overexpression. *P < 0.05, compared to the LV-con-RNAi or LV-con-ALK5 group at the same time point (Student’s t test). **f, g** The mNSS test and adhesive-removal somatosensory test were performed 3 d before and 1, 7, and 14 d after I/R (*n* = 10 biological replicates). There were no significant differences among the groups 1 d after I/R. However, there were significant differences among the groups 7 d and 14 d after I/R. *P < 0.05, compared to the LV-con-RNAi group at the same time point; ^△^*P* < 0.05, compared to the LV-con-ALK5 group at the same time point; ^#^*P* < 0.05, compared to the MCAO/R group at the same time point (ANOVA)

LV-ALK5-RNAi, LV-con-RNAi, LV-ALK5 or LV-con-ALK5 were injected into the ischemic cortex 2 weeks before MCAO/R. GFP carried by lentivirus was observed in the injected cortex 2 w after injection (Fig. 2a). The expression of ALK5 was significantly lower in the LV-ALK5-RNAi group than in the LV-con-RNAi group at 24 h and 14 d after MCAO/R (*P* < 0.05, Fig. 2b, c), indicating that LV-ALK5-RNAi had been successfully transduced into neurons and efficiently reduced the expression of ALK5. Moreover, the expression of ALK5 was significantly higher in the LV-ALK5 group than in the LV-con-ALK5 group (*P* < 0.05, Fig. 2d, e), indicating that LV-ALK5 was successfully transduced into neurons and efficiently overexpressed ALK5.

### ALK5 promotes neurological function recovery

The mNSS and adhesive-removal somatosensory tests were performed to evaluate neurological function 3 d before and 1, 7 and 14 d after MCAO/R. At baseline, no significant differences in neurobehavioral scores were detected among the groups. At 24 h after I/R, the neurobehavioral score of the LV-ALK5-RNAi group was slightly worse than that of the LV-con-RNAi group, and the score of the LV-ALK5 group was slightly better than that of the LV-con-ALK5 group. However, there were no significant differences among the groups 24 h after I/R. The LV-ALK5-treated group displayed better neurobehavioral recovery than did the other groups in both tests 7 d as well as 14 d after I/R. Notably, we observed a significant continuous improvement trend in functional recovery from 7 d to 14 d. These data suggested that ALK5 may play an important role in the long-term recovery of neurological function after I/R injury (*P* < 0.05, Fig. 2f, g).

### ALK5 mediates axonal regeneration and axonal reorganization

**Fig. 3:**
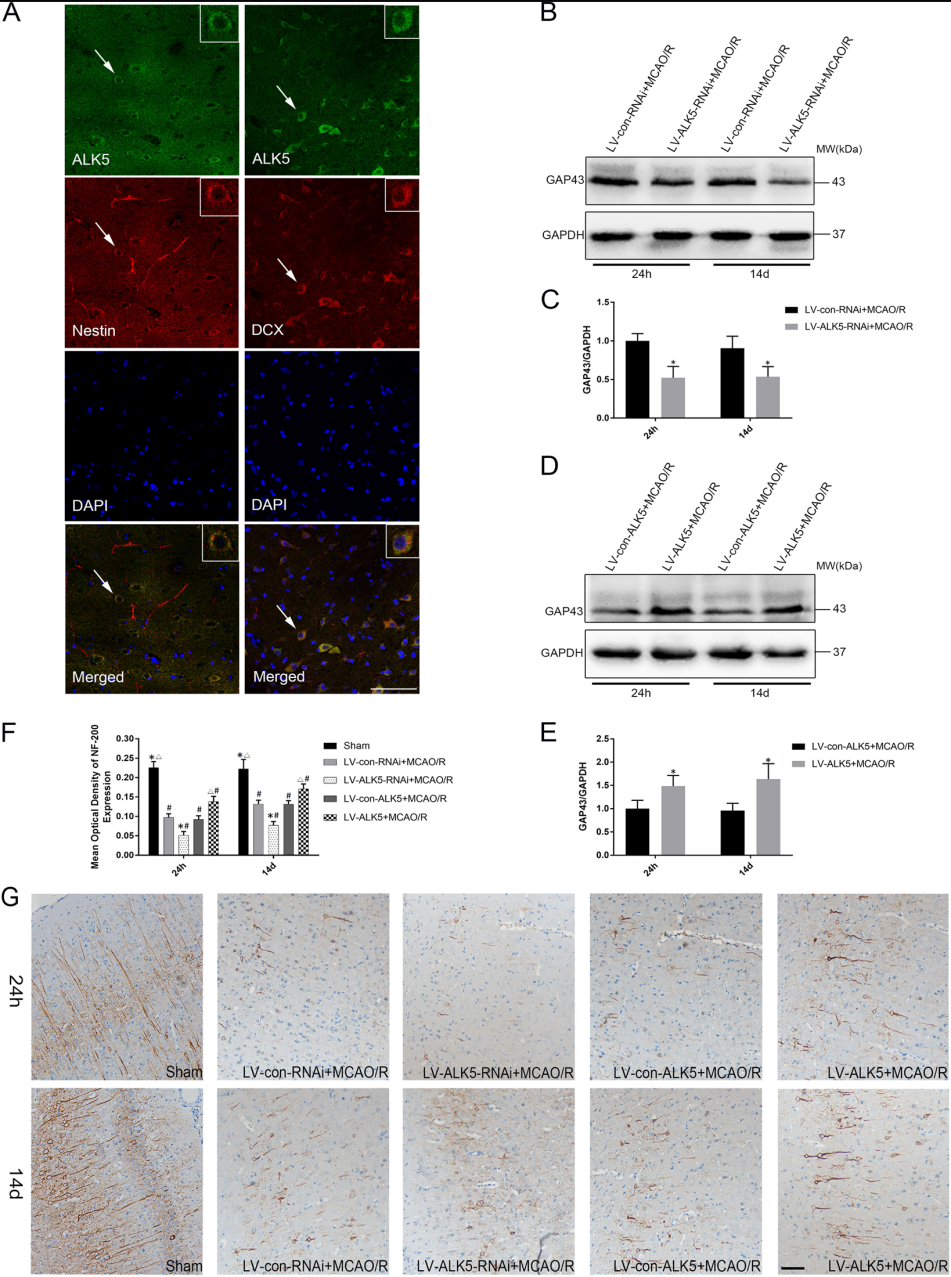
Colocalization of ALK5 with Nestin or DCX after I/R; ALK5 promotes axonal regeneration after I/R. **a** Representative images of immunofluorescence staining for ALK5 (green), Nestin (red)/ DCX (red) and cellular nuclei (blue). (scale bar = 100 μm). Arrows show the positive cells, and the inserted images show magnified images of representative cells. **b** Representative Western blot images of GAP-43 expression with LV-ALK5-RNAi or LV-con-RNAi injection 24 h and 14 d after I/R (*n* = 6 biological replicates). **c** Comparison of mean intensity ratios in Western blot analysis for GAP-43 with ALK5 knockdown. **d** Representative Western blot images of GAP-43 expression with LV-ALK5 or LV-con-ALK5 injection 24 h and 14 d after I/R (*n* = 6 biological replicates). **e** Comparison of mean intensity ratios in Western blot analysis for GAP-43 with ALK5 overexpression. *P < 0.05, compared to the LV-con-RNAi or LV-con-ALK5 group at the same time point (Student’s t test). **f** Comparison of the mean density value in immunohistochemical analysis for NF-200 expression. *P < 0.05, compared to the LV-con-RNAi group at the same time point; ^**△**^*P* < 0.05, compared to the LV-con-ALK5 group at the same time point; ^#^*P* < 0.05, compared to the sham group at the same time point (ANOVA). **g** Representative images of immunohistochemical staining for NF-200 expression in each group 24 h and 14 d after I/R (*n* = 5 biological replicates). (scale bar = 100 μm)

First, to investigate the role of ALK5 in neurogenesis after cerebral I/R injury, double immunofluorescence labeling was used to determine the relationship between ALK5, Nestin and DCX. After cerebral ischemia, ALK5 colocalized with Nestin (neural stem cell marker) and colocalized with DCX (neuroblast and immature neuron marker) (Fig. 3a). These results indicated that ALK5 may play a key role in neurogenesis after cerebral ischemia.

Then, axonal regeneration was assessed by upregulation of GAP-43 and NF-200. Western blot analysis showed that GAP-43 protein expression was lower in the LV-ALK5-RNAi group than in the LV-con-RNAi group at the same time point after MCAO/R (*P* < 0.05, Fig. 3b, c) and was higher in the LV-ALK5 group than in the LV-con-ALK5 group at both time points (*P* < 0.05, Fig. 3d, e). Immunohistochemical staining of NF-200 showed that the NF-200 positive fibers were long and neatly aligned in the sham groups and were short and disorderly after I/R injury. Compared with the MCAO/R groups, the LV-ALK5-RNAi group showed worsened NF-200 positive fibers, while the fibers in the LV-ALK5 group were better at 24 h after MCAO/R (*P* < 0.05, Fig. 3f, g). The recovery at 14 d was poor in the LV-ALK5-RNAi group and obvious in the LV-ALK5 group (*P* < 0.05, Fig. 3f, g).

**Fig. 4:**
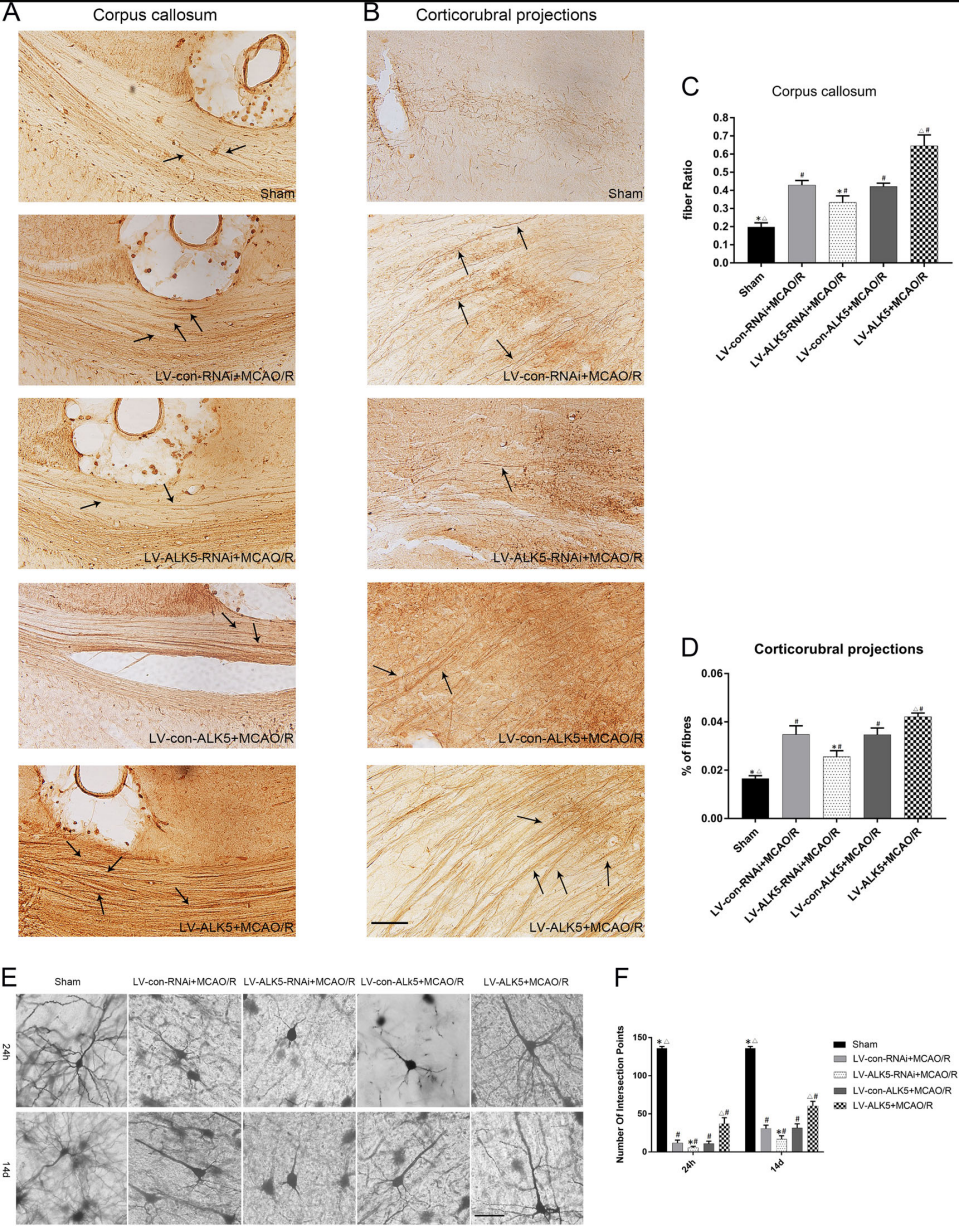
LK5 promotes BDA-positive fibers crossing the midline in the corpus callosum and red nucleus; ALK5 mediates dendritic plasticity after I/R. A **a** Representative images of BDA-positive fibers crossing the midline of the corpus callosum in each group (*n* = 5 biological replicates). **b** Representative images of BDA-positive corticorubral fibers crossing the midline to the right red nucleus in each group (*n* = 5 biological replicates). Arrows show the positive fibers. (scale bar = 100 μm). **c** Immunohistochemical analysis for BDA-positive fibers crossing the midline of the corpus callosum. **d** Immunohistochemical analysis for BDA-positive corticorubral fibers crossing the midline. *P < 0.05, compared to the LV-con-RNAi group; ^△^*P* < 0.05, compared to the LV-con-ALK5 group; ^#^*P* < 0.05, compared to the sham group (ANOVA). **e** Representative images of Golgi–Cox pyramidal neurons in each group 24 h and 14 d after I/R (*n* = 5 biological replicates). (scale bar = 100 μm). **f** Comparison of the number of intersection points in Sholl analysis for Golgi–Cox pyramidal neurons. *P < 0.05, compared to the LV-con-RNAi group at the same time point; ^△^*P* < 0.05, compared to the LV-con-ALK5 group at the same time point; ^#^*P* < 0.05, compared to the sham group at the same time point (ANOVA)

Axonal reorganization was measured by anterograde neuronal tracer BDA. First, we measured BDA-positive fibers crossing the midline of the corpus callosum in each group. Compared with the sham group, the MCAO groups showed significantly increased numbers of crossing midline fibers. The number in the LV-ALK5 group was much higher than that in the other four groups, while the number in the LV-ALK5-RNAi group was lower (*P* < 0.05, Fig. 4a, c). BDA-positive fibers could be observed in the denervated red nucleus in each group. The number of crossing midline corticorubral fibers was low in the sham group. LV-ALK5-RNAi-treated rats displayed a lower density of crossing midline corticorubral fibers than did the LV-con-RNAi-treated rats. Moreover, compared with the LV-con-ALK5 group, the LV-ALK5 group displayed a higher density of BDA-positive fibers crossing the midline (P < 0.05, Fig. 4b, d).

### ALK5 mediates dendritic plasticity

We performed Golgi–Cox staining to evaluate dendritic plasticity. First, we reconstructed the entire dendritic tree in two dimensions and measured the dendritic tracings using Sholl analysis. Sholl analysis showed that the length and distribution of dendrites were significantly decreased 24 h after MCAO/R. Additionally, the LV-ALK5-RNAi-treated group was worse than the LV-con-RNAi group, while the LV-ALK5 group was better than the LV-con-ALK5 group. At 14 d after MCAO/R, the recovery was poor in the LV-ALK5-RNAi group and obvious in the LV-ALK5 group (P < 0.05, Fig. 4e, f).

### ALK5 mediates Gadd45b expression by regulating the phosphorylation of Smad2/3, and ALk5 and Gadd45b are coimmunoprecipitated

**Fig. 5:**
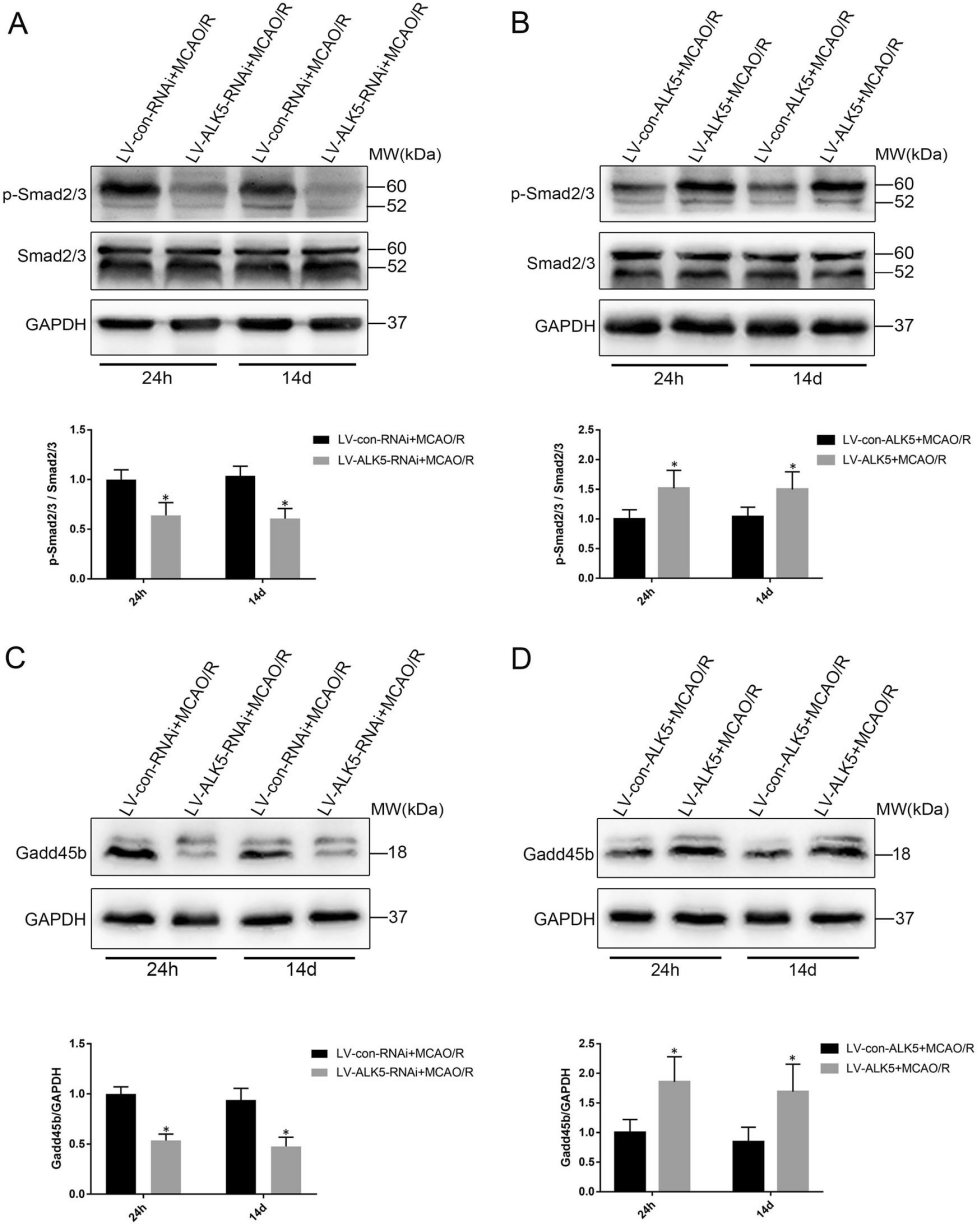
ALK5 regulates the phosphorylation of Smad2/3 and the protein levels of Gadd45b. **a** Representative Western blot images and bar graph summary of Smad2/3 phosphorylation with LV-ALK5-RNAi or LV-con-RNAi injection 24 h and 14 d after I/R (*n* = 6 biological replicates). **b** Representative Western blot images and bar graph summary of Smad2/3 phosphorylation with LV-ALK5 or LV-con-ALK5 injection 24 h and 14 d after I/R (*n* = 6 biological replicates). **c** Representative Western blot images and bar graph summary of Gadd45b expression with LV-ALK5-RNAi or LV-con-RNAi injection 24 h and 14 d after I/R (*n* = 6 biological replicates). **d** Representative Western blot images and bar graph summary of Gadd45b expression with LV-ALK5 or LV-con-ALK5 injection at 24 h and 14 d after I/R (*n* = 6 biological replicates). *P < 0.05, compared to the LV-con-RNAi or LV-con-ALK5 group at the same time point (Student’s t test)

**Fig. 6:**
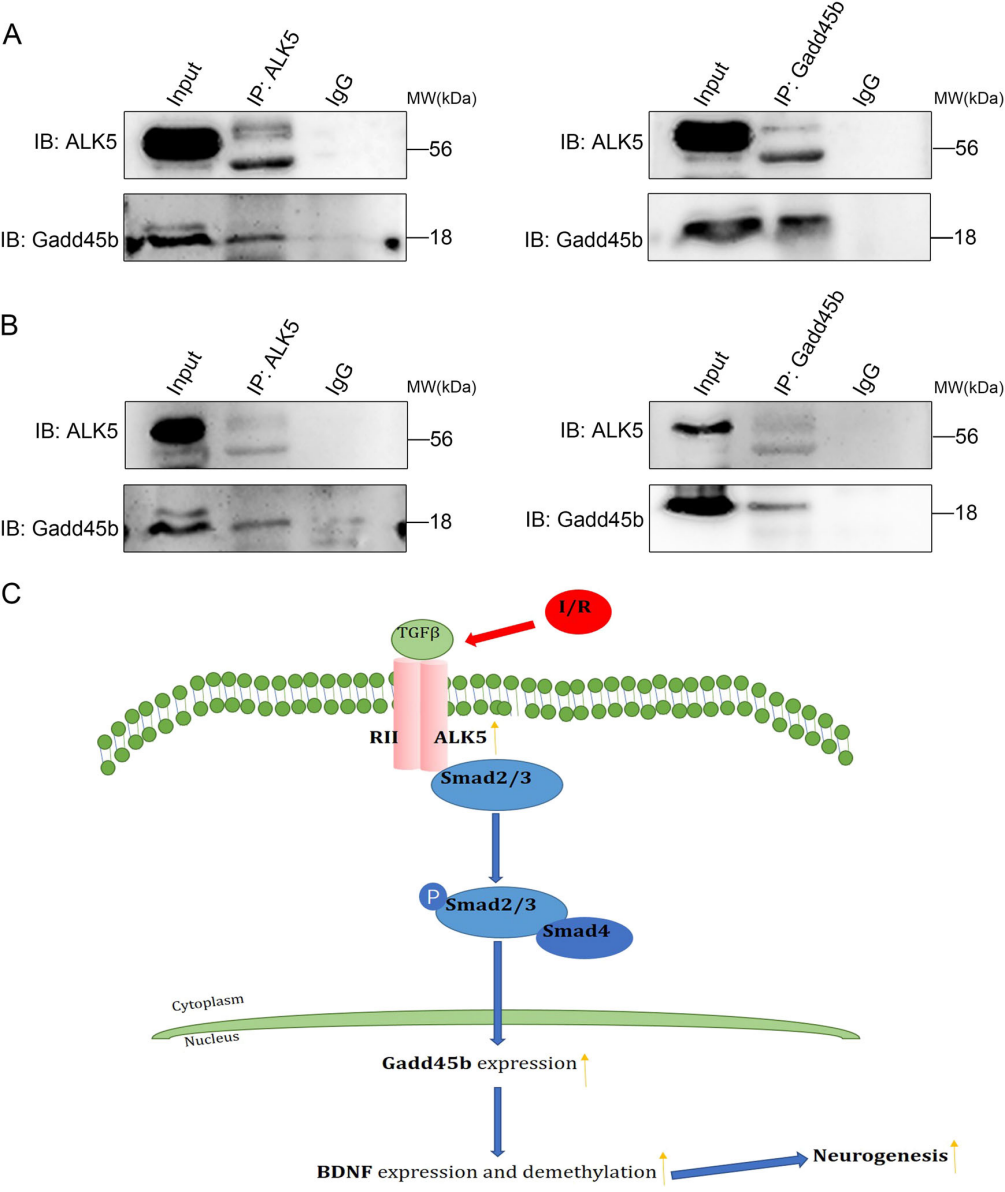
ALK5 interacts with Gadd45b after I/R; a schematic diagram depicting the possible mechanism of ALK5 signaling regulating neurogenesis after cerebral I/R. **a** Coimmunoprecipitation experiments of ALK5 and Gadd45b in the ischemic cortex 24 h after I/R. **b** Coimmunoprecipitation experiments of ALK5 and Gadd45b in the ischemic cortex 14 d after I/R. These results show that ALK5 coimmunoprecipitated with Gadd45b after cerebral I/R. **c** Ischemia activates ALK5, which catalyzes the phosphorylation of Smad2/3 and forms a complex with Smad4. In the nucleus, the complex promotes Gadd45b expression, which elevates the expression and demethylation of BDNF, thereby improving neurogenesis

In the canonical TGF-β signaling pathway, ALK5 can promote the phosphorylation of Smad2/3, which forms a heteromeric complex with Smad4 and translocates into the nucleus^9,12^. Additionally, ALk5/Smad2/3 may upregulate Gadd45b expression^18^. To determine whether ALK5/Smad2/3 mediates Gadd45b protein expression after I/R injury, we first examined the effect of LV-ALK5-RNAi or LV-ALK5 on the phosphorylation of Smad2/3 and expression of Gadd45b by Western blotting. After I/R injury, LV-ALK5-RNAi treatment significantly reduced the phosphorylation of Smad2/3 (*P* < 0.05, Fig. 5a) and the expression of Gadd45b (*P* < 0.05, Fig. 5c). Moreover, the phosphorylation of Smad2/3 (*P* < 0.05, Fig. 5b) and the protein levels of Gadd45b (*P* < 0.05, Fig. 5d) were markedly increased by ALK5 overexpression. To further verify the interaction between ALK5 and Gadd45b, we performed coimmunoprecipitation analysis 24 h and 14 d after MCAO/R. Based on the results, ALK5 interacted with Gadd45b in the ischemic cortex (Fig. 6a, b). Based on these data, we speculated that ALK5 can mediate Gadd45b protein expression by regulating the phosphorylation of Smad2/3.

## Discussion

Neurogenesis is the key to neurological function recovery after cerebral I/R injury. According to a previous study, ALK5 signaling is a major determinant of adult neurogenesis^12^. However, whether ALK5 signaling is involved in neurogenesis after cerebral ischemia and its mechanism is not fully understood. In this study, ALK5 signaling promoted neurological function recovery by mediating neurogenesis after cerebral I/R injury via the Smad2/Smad3/Gadd45b pathway.

ALK5 is the receptor of TGF-β and plays a pivotal role in the canonical TGF-β signaling pathway. The protein level of ALK5 is low in normal brain tissue, but the expression of ALK5 is significantly increased in brain injury (such as hypoxia, ischemia and poisoning)^15,28^. Consistent with previous studies, increased expression of ALK5 was observed in the ischemic cortex of a MCAO/R rat model, suggesting that ALK5 also plays a critical role in the pathological process of cerebral I/R injury. According to recent studies, ALK5 signaling seems to be involved in neurological function recovery^29,30^. In line with these studies, in our study, blockade of ALK5 expression caused poor neurological function recovery after I/R injury, while overexpression of ALK5 promoted functional recovery. However, the mechanism is not very clear. Neurological recovery therapy based on neuroplasticity plays a critical role in functional recovery after cerebral ischemia^31,32^. At the cellular level, neuroplasticity is manifested by changes in dendritic branches, axonal sprouts, dendritic spine density, synapse number, and receptor density^33^. Axonal remodeling and dendritic regulation in the ipsilateral and contralateral cortex in cerebral infarction are the key factors for the repair of neural structure and the recovery of spontaneous neural function after cerebral ischemia^34^. Moreover, the commissural fibers across the corpus callosum undergo remodeling and axonal sprouting^35^. Through these structural changes, neural control of denervated tissue can be reconstructed, which is the basis of functional reconstruction after nervous system injury^36^. Double immunofluorescence staining revealed that ALK5 was colocalized with Nestin as well as DCX. Nestin is a neural stem cell marker, and DCX is an immature neuron marker. These results suggest that ALK5 signaling is essential for neurogenesis.

To investigate whether ALK5 promotes neurological function recovery by affecting neuroplasticity, we focused on changes in axonal plasticity and dendritic plasticity. Axonal plasticity including axonal reorganization and axonal regeneration^37^. Axonal regeneration is reflected by upregulated expression of GAP-43 and NF-200. GAP-43 is a pivotal component of the regenerative response in the nervous system and is present at high levels in neuronal growth cones during axonal regeneration. NF-200 is a major component of neuronal cytoskeletons and can reflect neuronal function and axonal regeneration. For axonal reorganization, we focused on BDA-positive fibers crossing the midline of the corpus callosum and corticorubral fibers originating from the contralateral sensorimotor cortex. Dendritic plasticity can be identified by Golgi–Cox staining with Sholl analysis. An increase in dendritic branches and dendritic length occurs after ischemic stroke^38^. As demonstrated in the present study, the overexpression or knockdown of ALK5 enhanced or reduced axonal plasticity and dendritic plasticity, respectively, indicating that ALK5 signaling mediates neurogenesis after cerebral I/R injury. However, a study suggested that ALK5 is involved in glial scar formation, thereby inhibiting neurological function recovery^39^. We speculate that this opposing study conclusion may be related to the important roles of ALK5 in multiple signaling and functional pathways. Therefore, we need to further investigate the mechanism of ALK5 signaling that mediates neurogenesis and find a more effective therapeutic strategy for neurological function recovery.

The Gadd45 family includes three members, Gadd45a, Gadd45b, and Gadd45y; of these family members, Gadd45b is the only member that can be regulated by TGF-β^40^. Gadd45b has been implicated as a stress-response gene for physiological or environmental stressors; it influences apoptosis, cell-cycle arrest, cell growth and DNA repair^41,42^. In our previous study, Gadd45b mRNA and protein were significantly increased after I/R^43^. Gadd45b knockout mice display a decrease in the expression and DNA demethylation of BDNF^17^. Additionally, our previous studies suggested that Gadd45b knockdown reduces the expression and demethylation of BDNF, with a concomitant decrease in neurological function recovery; we also speculate that Gadd45b promotes axonal plasticity by promoting the expression and DNA demethylation of BDNF^7^. The corticospinal tract (CST) is the direct central nervous structure that controls limb movement. The direct injury of CST caused by cerebral infarction is the direct neurological basis of motor dysfunction. The ipsilateral or contralateral CST undamaged axons regenerate and sprout to form lateral branches and reconstruct the neural control of the anterior horn cells of the spinal cord, which is the material basis for limb motor function rehabilitation^44,45^. In the present study, changes in Gadd45b expression were consistent with ALK5 expression and corresponded to changes in neural plasticity and neurological function recovery. Based on these data, we believe that ALK5 acts upstream of Gadd45b, which affects the protein levels and demethylation of BDNF by regulating the expression of Gadd45b, thereby promoting functional recovery.

Based on all our findings, we propose the following possible mechanisms of ALK5-regulated neurological recovery after cerebral I/R injury. The activation of ALK5 signaling by I/R promotes the phosphorylation of Smad2/3, which form a heteromeric complex with Smad4 and translocate into the nucleus where they interact with Gadd45b (Fig. 6c). Notably, although Gadd45b can be regulated by ALK5 signaling, ALK5 is not the only factor that can regulate it^42,46^.

In conclusion, our findings suggest that ALK5 signaling may be an important mediator of neurogenesis and functional recovery after cerebral I/R injury. Although the mechanisms require more in-depth investigation, our present study could provide insight into the role of ALK5 signaling in cerebral I/R injury. Targeting ALK5 may be a potential therapeutic strategy for cerebral I/R injury.
